# A Digital Cognitive-Physical Intervention for Attention-Deficit/Hyperactivity Disorder: Randomized Controlled Trial

**DOI:** 10.2196/55569

**Published:** 2024-05-10

**Authors:** Licong Zhao, Heather Agazzi, Yasong Du, Hongdao Meng, Renya Maku, Ke Li, Peter Aspinall, Cynthia Wilson Garvan, Shuanfeng Fang

**Affiliations:** 1 Department of Child Healthcare Children’s Hospital Affiliated to Zhengzhou University Zhengzhou China; 2 Department of Pediatrics & Department of Psychiatry and Behavioral Neurosciences College of Medicine University of South Florida Tampa, FL United States; 3 Department of Child & Adolescent Psychiatry Shanghai Mental Health Center Shanghai Jiaotong University Shanghai China; 4 College of Behavioral & Community Sciences University of South Florida Tampa, FL United States; 5 College of Public Health University of South Florida Tampa, FL United States; 6 Recovery Plus Chengdu China; 7 Department of Anesthesiology College of Medicine University of Florida Tampa, FL United States

**Keywords:** school-age children, cognitive training, exercise therapy, gamification, ADHD, attention deficit, attention-deficit/hyperactivity disorder, RCT, randomized controlled trial, executive function, digital intervention, AR, augmented reality

## Abstract

**Background:**

Attention-deficit/hyperactivity disorder (ADHD) is one of the most common neurodevelopmental disorders among children. Pharmacotherapy has been the primary treatment for ADHD, supplemented by behavioral interventions. Digital and exercise interventions are promising nonpharmacologic approaches for enhancing the physical and psychological health of children with ADHD. However, the combined impact of digital and exercise therapies remains unclear.

**Objective:**

The aim of this study was to determine whether BrainFit, a novel digital intervention combining gamified cognitive and exercise training, is efficacious in reducing ADHD symptoms and executive function (EF) among school-aged children with ADHD.

**Methods:**

This 4-week prospective randomized controlled trial included 90 children (6-12 years old) who visited the ADHD outpatient clinic and met the diagnostic criteria for ADHD. The participants were randomized (1:1) to the BrainFit intervention (n=44) or a waitlist control (n=46) between March and August 2022. The intervention consisted of 12 30-minute sessions delivered on an iPad over 4 weeks with 3 sessions per week (Monday, Wednesday, and Friday after school) under the supervision of trained staff. The primary outcomes were parent-rated symptoms of attention and hyperactivity assessed according to the Swanson, Nolan, and Pelham questionnaire (SNAP-IV) rating scale and EF skills assessed by the Behavior Rating Inventory of Executive Function (BRIEF) scale, evaluated pre and post intervention. Intention-to-treat analysis was performed on 80 children after attrition. A nonparametric resampling-based permutation test was used for hypothesis testing of intervention effects.

**Results:**

Among the 145 children who met the inclusion criteria, 90 consented and were randomized; ultimately, 80 (88.9%) children completed the study and were included in the analysis. The participants’ average age was 8.4 (SD 1.3) years, including 63 (78.8%) male participants. The most common ADHD subtype was hyperactive/impulsive (54/80, 68%) and 23 (29%) children had severe symptoms. At the endpoint of the study, the BrainFit intervention group had a significantly larger improvement in total ADHD symptoms (SNAP-IV total score) as compared to those in the control group (β=–12.203, 95% CI –17.882 to –6.523; *P*<.001), owing to lower scores on the subscales Inattention (β=–3.966, 95% CI –6.285 to –1.647; *P*<.001), Hyperactivity/Impulsivity (β=–5.735, 95% CI –8.334 to –3.137; *P*<.001), and Oppositional Defiant Disorder (β=–2.995, 95% CI –4.857 to –1.132; *P*=.002). The intervention was associated with significant reduction in the Metacognition Index (β=–6.312, 95% CI –10.973 to –1.650; *P*=.006) and Global Executive Composite (β=–5.952, 95% CI –10.214 to –1.690; *P*=.003) on the BRIEF. No severe intervention-related adverse events were reported.

**Conclusions:**

This novel digital cognitive-physical intervention was efficacious in school-age children with ADHD. A larger multicenter effectiveness trial with longer follow-up is warranted to confirm these findings and to assess the durability of treatment effects.

**Trial Registration:**

Chinese Clinical Trial Register ChiCTR2300070521; https://www.chictr.org.cn/showproj.html?proj=177806

## Introduction

Attention-deficit/hyperactivity disorder (ADHD) is the most common neurodevelopmental disorder among school-age children [1], with an estimated prevalence of 8.9% in the United States [2] and 6.5% in China [3,4]. The core symptoms of ADHD are characterized by inattention, impulsivity, and/or hyperactivity, and these symptoms lead to functional impairment across multiple settings [5]. These symptoms often co-occur with psychological disorders (eg, disruptive behavior, conduct disorder, anxiety, and depression). Despite the heavy disease burden of ADHD and long-term negative outcomes (eg, substance use disorders, reduced employment and income, and poor social outcomes) [6], many youth with ADHD do not access treatment to mediate the substantial impact of these disorders on daily functioning.

The first-line treatment for ADHD traditionally includes pharmacotherapy, often complemented by nonpharmacological behavioral interventions [7]. In addition, most children with ADHD benefit from school-based interventions that target on-task behavior, academic skills, and social interactions with peers [8]. While pharmacotherapy has proven to be effective for the short-term treatment of ADHD symptoms and approximately two-thirds of children with a current ADHD diagnosis have taken prescribed medication [3], adherence is affected by patient and caregiver attitudes about medication effectiveness and practical barriers. A study on medication adherence in children, adolescents, and adults with ADHD found that the main factors contributing to poor medication adherence included fear of addiction and doubts about the effectiveness of the medication [9]. In addition, some young people with ADHD stop taking their medication due to the challenges associated with accessing the prescription, including committing to medical appointments every 3 months for refills, paying for the cost of the prescription, the time and cost of missing work and attending doctor visits, and finding new providers when transitioning from pediatric to adult care [10]. Thus, even though medication can improve ADHD symptoms in the short term, many people with ADHD perceive the costs of pharmacotherapy to outweigh the benefits.

Behavioral interventions are recommended as the first-line treatment for ADHD in preschool-aged children (4-5 years) [11]. Indeed, behavioral interventions play an important role in the development of organizational skills and executive function (EF) [12]. These interventions include self-monitoring, positive reinforcement for on-task behavior, a token economy, and chunking tasks while providing frequent feedback on performance [13]. These interventions are often taught in the context of parent management training, which has long been considered a gold-standard intervention to address disruptive behaviors in children [14]. Behavioral interventions for ADHD are important because youth learn coping strategies to improve their developmental trajectory across academic, social, and employment settings, potentially reducing overall functional impairment. In addition, behavioral interventions do not carry the same side effects of medication intervention. While behavioral interventions are very effective for treating symptoms of ADHD, less than half of children with a current diagnosis were reported to have received such treatment before 2019 in the United States [15]. The treatment rates could be much lower in less-developed countries and in rural regions of developed countries. In addition, behavioral and psychosocial interventions require extensive time and dedication of caregivers, often resulting in low adherence rates [16,17]. As with medication intervention, there are barriers to accessing behavioral interventions, including a limited number of providers, limited providers who accept Medicaid insurance in the United States, limited availability of treatment in rural settings, long wait lists, and cash-only services [18].

Digital treatment for ADHD may provide an alternative to address some of the challenges in accessing ADHD services with fewer side effects and a lower likelihood of addiction [19]. Digital interventions that can be delivered via mobile phones, tablets, or web-based platforms may provide a mechanism for behavioral interventions to be more accessible, better integrated across multiple settings (eg, home, school, and health services), and enable individuals (and families) to take care of themselves. Some research has shown that cognitive training targeting ADHD symptoms such as attention, response inhibition, and working memory can be designed to engage users through a computer or mobile phone portal [20,21]. A meta-analysis showed that although cognitive training does improve performance related to some neurophysiological functions, the effect on ADHD symptoms is not yet known [22]. Notably, interventions that included multiple cognitive processes had higher effect sizes than those of training that targeted only a single cognitive function [23].

Emerging evidence indicates that digital therapies can affect brain functions such as increased working memory, processing speed, and concentration, and these treatments were proven to have therapeutic effects in a variety of mental and behavioral disorders [24,25]. Kollins et al [19] developed a digital therapy named AKL-T01, which had a beneficial effect on the average attention measure of children with ADHD compared to that of the control group. For its first-in-class breakthrough, the resulting digital intervention has received approval for clinical use by the US Food and Drug Administration (FDA). These findings suggest that digital therapy may be effective in the nonpharmacological management of ADHD symptoms. Digital therapies have the potential to be conducted at any time or place, which could alleviate the burden on caregivers to organize interventions for their children with ADHD. In turn, this may improve patient adherence to the intervention and lead to lasting therapeutic effects. Importantly, digital therapies do not require substantial professional human resources, which could benefit many less-developed countries and regions.

As a nonpharmacologic therapy, exercise has been shown to improve the physical and psychological health of children with ADHD. In recent years, exercise has been linked to positive neuropsychiatric outcomes such as neurocognitive and executive functions [17,26,27]. For example, a meta-analysis showed that exercise therapy can improve core symptoms and EF in children and adolescents with ADHD, especially with closed-skill training [28]. Based on the advantages of digital therapy and exercise therapy in improving ADHD, Benzing et al [25] developed a digital exercise game and conducted an 8-week intervention test on children with ADHD. The results suggested improvements in EF (eg, reaction time in inhibition and switching) when compared to the control group, as well general improvements in psychopathology and motor abilities. However, evidence regarding whether digital behavioral interventions in conjunction with exercise therapy would confer clinical benefits above and beyond those obtained when each intervention is used separately remains limited.

**Figure 1:**
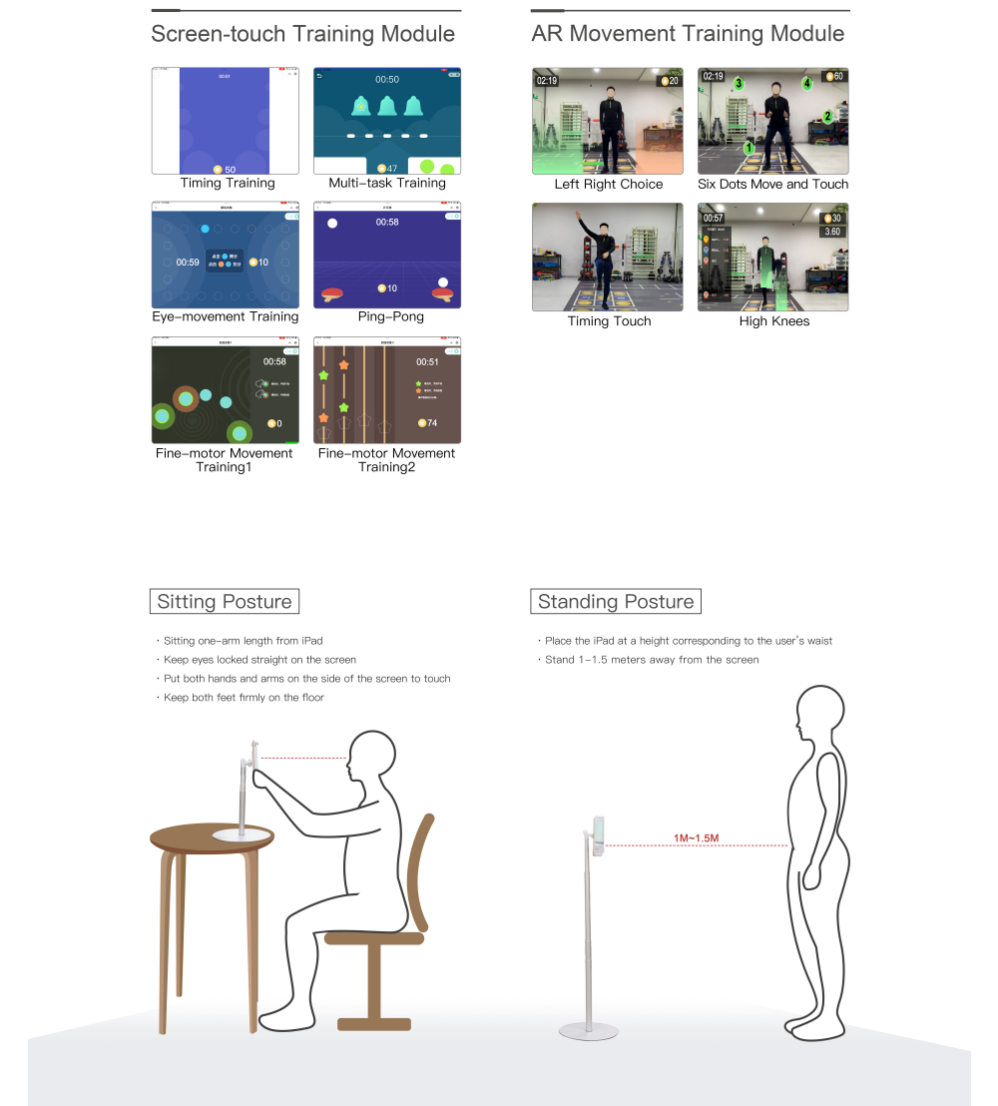
Illustrative example of Brainfit setup and modules. AR: augmented reality.

BrainFit is an interactive augmented reality (AR)–based exercise therapy designed and executed in a digital form that uses a pioneering engine to evaluate, develop, and dynamically optimize skills and abilities in a hierarchical manner. BrainFit was created to improve concentration and other EF skills that are typically impaired in youth with ADHD. A variety of training paradigms integrate digital intervention modules through the medium of touchscreen interaction and AR motion interaction. Figure 1 shows the information on BrainFit example modules. The adaptive training algorithm modifies the levels of task difficulty based on the task performance of the user and thus optimizes engagement.

The purpose of this randomized controlled trial was to examine whether a novel digital intervention combining gamified cognitive training and exercise (BrainFit) would be effective in treating ADHD symptoms among children with ADHD aged 6 to 12 years, as well as to evaluate whether any beneficial effects on the core ADHD symptoms and EF could be observed.

## Methods

### Study Design

This study was an open-label, randomized, parallel-group trial. The trial was conducted at the Children’s Hospital Affiliated to Zhengzhou University (Zhengzhou, Henan, China) from March to August of 2022. All participants were screened for eligibility before being randomly assigned to the BrainFit intervention or control group.

### Participants

The participants were recruited from Children’s Hospital Affiliated to Zhengzhou University. The inclusion criteria were: (1) aged 6-12 years; (2) ADHD diagnosed by clinicians based on *The Diagnostic and Statistical Manual of Mental Disorders*, *5th edition* (DSM-5) [29]; and (3) an IQ score >80, measured by the validated Chinese version of the Wechsler Intelligence Scale for Children, Fourth Edition. Exclusion criteria were: (1) presence of comorbid severe psychiatric or physical disorders that may affect attention (eg, autism spectrum disorder, learning disabilities, abnormal blood glucose, anemia, hyperthyroidism, and hyperadrenocorticism) and (2) current or recent (within the last 6 months) medication treatment (eg, methylphenidate, atomoxetine, risperidone, modafinil) or (3) behavioral treatment for ADHD.

### Randomization

Prior to randomization, participants were informed that they would either be assigned to the 4-week intervention group or the waitlist control group. The randomization schedule was generated by an independent statistician not involved in the clinical trial (using the PROC PLAN procedure in SAS 9.4) and the randomization schema was reproducible. Participants were not blinded to group assignment due to the nature of the digital intervention.

### Equipment and Staff Training

The intervention was delivered via iPads preinstalled with the BrainFit software.

Two researchers received training on intervention delivery using BrainFit, which included reviewing the intervention protocol and standardized training manual, practicing intervention delivery steps, and understanding how to respond to potential adverse events. The training material also included a tutorial for BrainFit and a written overview of the study protocol.

### Intervention Overview

At the time of diagnosis, participants’ parents were provided psychoeducation on ADHD according to Chinese consensus standards for ADHD treatment [30]. The intervention was facilitated by trained researchers and consisted of 12 sessions (30 minutes each) delivered via iPad technology over 4 weeks. Each participant received three sessions per week (Monday, Wednesday, and Friday after school) with appropriate training levels tailored to their individual conditions. The intervention included six modules, delivered in the same sequence, with adaptive difficulty levels to increase engagement. Participants earned scores based on completing tasks correctly and on time, and games were upgraded or downgraded based on their performance. Participants and their caregivers received study session reminders regularly to reduce missing data, missed sessions, and attrition. Participants in the control group received no intervention during the active study period other than the previously mentioned psychoeducation.

### Measures

Demographic data were collected from caregivers, including the caregiver report on the child’s social skills. ADHD symptoms and EF were assessed as primary outcomes at baseline and at 4 weeks post randomization by experienced trainers. Demographic data included sex (female or male), age (years), school report card–based academic performance (quartiles), living arrangement (living with parents or living with parents and grandparents), parent-rated sociability (low, medium, or high), and referral source (ie, teacher, parent, physician, or other). ADHD subtype and severity were defined according to the DSM-5. ADHD symptoms were assessed using the Chinese version of the Swanson, Nolan, and Pelham Questionnaire (SNAP-IV) 26-item parent rating scale [31], which has satisfactory measurement properties regarding test-retest reliability and validity. The SNAP-IV measures ADHD inattention symptoms (items 1-9) and symptoms of hyperactivity/impulsivity (items 10-18). In addition, items 19-26 assess symptoms of oppositional defiant disorder (ODD). Each item is ranked on a 4-point scale (0=“not at all,” 2=“just a little,” 3=“quite a bit,” and 4=“very much”). SNAP-IV is one of the most common methods measuring ADHD symptom severity, in which a higher score indicates more severe symptoms. The Chinese version of the Behavior Rating Inventory of Executive Function (BRIEF) parent form was used to assess EF [32]. The BRIEF has 86 items with each item rated on a 3-point scale (1=“never,” 2=“sometimes,” and 3=“often”). The BRIEF yields an overall Global Executive Composite (GEC) score, which is composed of two indices: the Behavioral Regulation Index (BRI) and Metacognition Index (MI). The BRI has three clinical scales (Inhibit, Shift, and Emotional Control) and the MI is comprised of five clinical scales (Initiate, Shift, Memory, Plan/Organize, Monitor, and Organization of Materials). The BRIEF has good psychometric properties, including test-retest reliability (intraclass correlation=0.68-0.89), internal consistency (Cronbach α=0.74-0.96), and validity (Pearson correlation coefficient=0.41-0.64) [33]; higher scores indicate worse EF.

### Sample Size

In a review of 21 intervention studies and their effect on EF in ADHD, a medium effect size was noted (standard mean difference [SMD] 0.611) [34]. We hypothesized a similar effect in the current study. Power analyses determined that a sample size of at least 30 participants per arm would be sufficient to achieve power of 80% with a Type I error rate of .05. Assuming a 20% dropout rate, a total sample of 80 participants was needed (40 per group).

### Adverse Events Reporting

Adverse events were assessed at each session. Participants could also report adverse events by calling a study telephone line. All researchers were trained to record details of any adverse events and a participant would be followed until the event resolved. All potential adverse events were reported and reviewed by the principal investigator. One adverse event was reported wherein a legal guardian withdrew a participant from the study after three sessions due to a fever; this event is unlikely to be related to the intervention.

### Statistical Analysis

We used IBM SPSS Statistics for Windows version 23.0 and Stata BE version 17 for all data processing and statistical analyses. We present continuous data as mean (SD) and categorical data as numbers and percentages. To assess any potential differences in participant demographic variables between the intervention and control groups, we used the independent-sample *t* test and Mann-Whitney *U* test for continuous variables and ordinal variables; χ^2^ tests were used for comparisons of categorical data. For the multivariable regression analysis of the primary outcomes, SNAP-IV and BRIEF scores were regressed onto the independent variables (treated as dummy variables), including age, sex, whether the participant was the first child, ADHD subtype, academic performance, and sociability ratings. These independent variables were selected based on clinical judgment and the literature. Controlling for these baseline characteristics in the regression model can generally increase the precision of the estimator for the average treatment effect [35]. All independent variables were entered into the model simultaneously. To obtain unbiased intervention effect estimates and quantify the uncertainty surrounding the point estimates, we used the Fisher-Pitman permutation test for two independent samples. This permutation test is a powerful alternative to the Wilcoxon-Mann-Whitney rank-sum test [36,37]. This approach has the benefit of making no assumptions about the distributional properties of the joint distributions of variables as well as minimizing the false-positive results due to multiple testing with traditional regression-based hypothesis testing [38,39]. Because the exact calculation of the test can be extremely computationally intensive, a Monte Carlo–based algorithm (with 10,000 repetitions) was used to increase efficiency with minimal loss of accuracy. We used an α level of .05 for determining statistical significance. The effect size (Cohen *d*) for each outcome measure was calculated with 95% CIs based on the nonparametric bootstrap method (with 5000 repetitions) to quantify the uncertainty surrounding the point estimates [40].

### Ethical Considerations

The study protocol was approved by the Medical Ethics Committee of Henan Children’s Hospital (2021-H-H09) before study initiation. The legal guardians of all participants provided written informed consent and children provided assent to participate before baseline assessment. All families were informed that participation was voluntary, that data collected during the study will be kept confidential, and that they can withdraw their consent at any time without negative consequences to their care. Each participant received ¥600 (US $82.80) as compensation for completing all sessions of the intervention. If a participant completed all assessments, the various scale assessments were offered at no cost to the participants. They also receive an explanatory document and consent form from the researchers that included contact information for any inquiries. The personal information of the participants and their guardians was anonymized, and a unique study ID number was assigned to each participant for data entry, management, and analysis. The individual pictured in Figure 1 provided written informed consent to allow their image to be published.

## Results

### Participants

**Figure 2:**
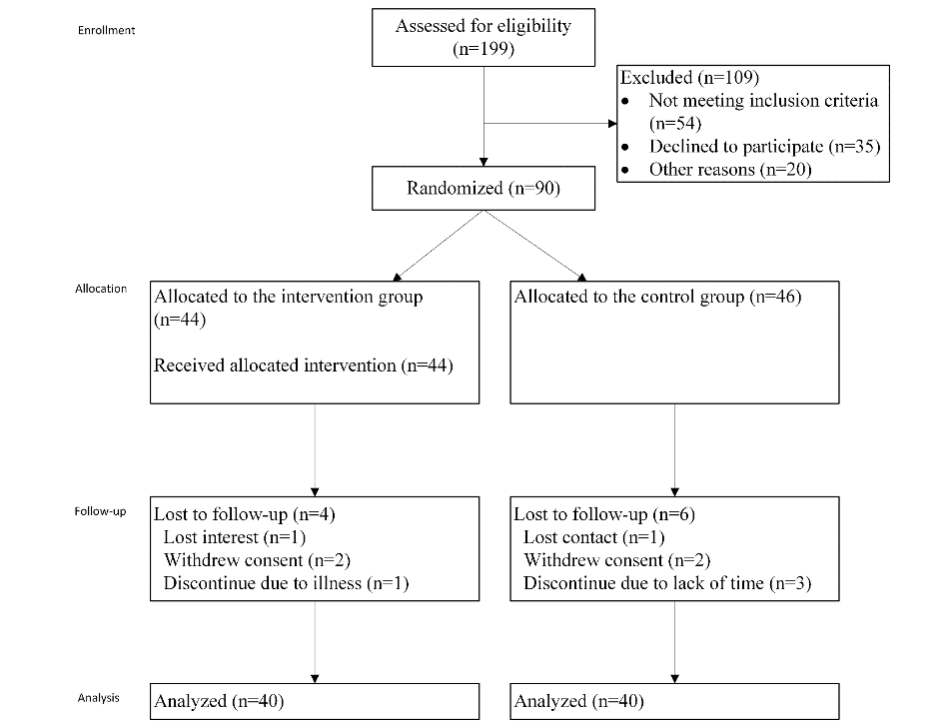
CONSORT (Consolidated Standards for Reporting Trials) diagram of participation flow in the study.

A total of 199 children were assessed for eligibility and 145 were deemed to be eligible. Among the eligible candidates, 90 children were randomized into either the intervention (n=44) or control (n=46) group. Eighty children completed the study and their data were analyzed, as described in the CONSORT (Consolidated Standards for Reporting Trials) diagram in Figure 2.

Table 1 summarizes the demographic characteristics of participants. The participants’ mean age was 8.4 years, including 63 (78.8%) male participants. The participants’ mean IQ was 98.7 (SD 10.8). The academic performance of more than 65% of the participants with ADHD ranked in the bottom half of the class. Nearly 90% of the participants were rated as having poor social skills by their caregivers and nearly 80% were the first child in the family. More than 60% had a nuclear family model (ie, living with parents only) and were referred to the hospital by a family member. Age, sex, IQ, achievement, social skills, ADHD severity, and prevalence of each ADHD subtype were not significantly different between the two groups of participants (all *P*>.05).

The primary outcome was ADHD symptoms as measured by the SNAP-IV total and subscale scores. Table 2 shows the results of the parents’ rating of SNAP-IV scores at baseline and at the study endpoint in the intervention and control groups. Participants in the intervention group had a significantly larger reduction in SNAP-IV total scores than those in the control group. For subscale scores, the overall reduction was attributable to significantly lower scores on the Inattention, Hyperactivity/Impulsivity, and ODD subscale scores.

In terms of EF as rated by the BRIEF, participants in the intervention group had a significantly larger reduction on the MI and the GEC, with a smaller and nonsignificant reduction in the BRI (Table 2). Except for the Inhibit clinical scale, children in the intervention group showed a larger reduction across all clinical scales as compared to those in the control group, with five clinical scales showing significant decreases: Initiate, Working Memory, Plan/Organize, Organization of Materials, and Monitor. The Initiate clinical scale decreased from a mean of 63.10 to 54.25 for participants in the intervention group, whereas the control group scores decreased from 59.90 to 59.03 (β=–5.80, *P*=.005).

**Figure 3:**
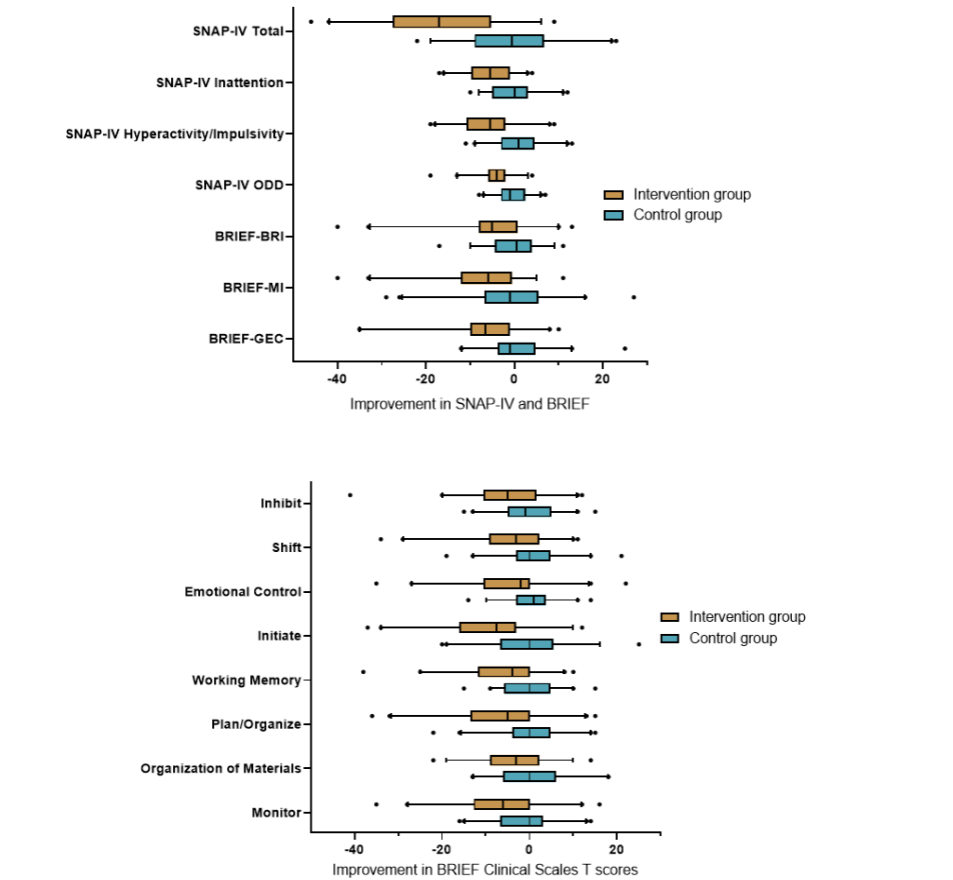
Box plots of changes in SNAP-IV and BRIEF T scores over 4 weeks according to treatment group. BRIEF: Behavior Rating Inventory of Executive Function; BRI: Behavioral Regulation Index; GEC: Global Executive Composite; MI: Metacognition Index; ODD: oppositional defiant disorder; SNAP-IV: Swanson, Nolan, and Pelham questionnaire.

Figure 3 depicts the improvements in the context of the wide range of baseline scores for all participants. The effect sizes were generally small to moderate for the outcomes that showed a statistically significant difference in improvements between the intervention and control groups.

**Table 1:** Baseline characteristics of the study sample overall and in the study groups allocated by random assignment.

Variables		Overall (N=80)	Intervention group (n=40)	Control group (n=40)	*P* value	
Age (years), mean (SD)		8.4 (1.3)	8.5 (1.5)	8.3 (1.1)	.43^a^	
Boys, n (%)		63 (79)	34 (85)	29 (73)	.27^b^	
IQ, mean (SD)		98.7 (10.8)	98.4 (10.6)	99.0 (10.6)	.81^a^	
**Academic performance, n (%)**						.43^a^
	Top quartile	9 (11)	6 (15)	3 (8)		
	2nd quartile	16 (20)	5 (13)	11 (28)		
	3rd quartile	34 (43)	22 (55)	12 (30)		
	Bottom quartile	21 (26)	7 (18)	14 (35)		
**Parent-rated** **sociability, n (%)**						.65^a^
	Low	29 (36)	16 (40)	13 (33)		
	Medium	44 (55)	19 (48)	25 (63)		
	High	7 (9)	5 (13)	2 (5)		
First child, n (%)		66 (83)	35 (88)	31 (78)	.24^b^	
Living with parents only, n (%)		48 (60)	25 (63)	23 (58)	.65^b^	
**Family income, n (%)**						.88^a^
	Below average	12 (15)	6 (15)	6 (15)		
	Average	53 (66)	27 (68)	26 (65)		
	Above average	15 (19)	7 (18)	8 (20)		
**ADHD^c^ subtype, n (%)**						.14^a^
	Inattentive	5 (6)	4 (10)	1 (3）		
	Hyperactive/impulsive	54 (68)	23 (58)	31 (78)		
	Combined presentation	21 (26)	13 (33)	8 (20)		
**ADHD severity, n (%)**						.82^a^
	Mild	25 (31)	12 (30)	13 (33)		
	Moderate	32 (40)	16 (40)	12 (40)		
	Severe	23 (29)	12 (30)	11 (28)		
**Referral source, n (%)**						.63^a^
	Teacher	7 (9)	4 (10)	3 (8)		
	Physician	10 (13)	4 (10)	5 (15)		
	Family member	56 (70)	27 (68)	29 (73)		
	Other	7 (9)	5 (13)	2 (5)		

^a^Mann-Whitney *U* test.

^b^χ^2^ test.

^c^ADHD: attention-deficit/hyperactivity disorder.

**Table 2:** BrainFit app intervention effect estimates as compared to the control group.

Measure			Intervention group, mean (SD)				Control group, mean (SD)				Coefficient estimate^a^		Effect size (Cohen *d*) (95% CI)^b^		*P* value^c^
			Baseline		Endpoint		Baseline		Endpoint						
**SNAP-IV^d^**															
	Total	45.83 (13.32)		29.38 (11.87)		36.93 (12.44)		36.65 (12.75)		–12.203		1.258 (0.804-1.712)		<.001	
	Inattention	18.30 (4.83)		12.73 (4.77)		16.43 (2.18)		16.13 (5.10)		–3.966		0.986 (0.542-1.430)		<.001	
	Hyperactivity/Impulsivity	16.00 (6.29)		10.00 (5.49)		11.93 (5.74)		12.60 (5.89)		–5.735		1.107 (0.601-1.612)		<.001	
	ODD^e^	11.55 (5.10)		7.40 (5.10)		8.58 (4.96)		7.93 (4.81)		–2.995		0.894 (0.475-1.314)		.002^c^	
**BRIEF^f^ domain scores**															
	BRI^g^	57.63 (11.94)		52.15 (10.79)		51.88 (9.48)		51.98 (10.43)		–2.905		0.638 (0.230-1.047)		.13	
	MI^h^	65.63 (9.30)		57.55 (11.52)		64.40 (11.60)		63.68 (10.93)		–6.312		0.688 (0.251-1.126)		.006	
	GEC^i^	63.23 (10.03)		55.73 (11.42)		58.50 (10.00)		58.90 (10.16)		–5.952		0.856 (0.476-1.235)		.003	

^a^Multivariable linear regression model controlling for age, sex, being the first child, attention-deficit/hyperactivity disorder subtypes, academic performance, and social abilities.

^b^95% CIs were calculated based on the nonparametric bootstrap method (with 1000 repetitions).

^c^*P* values were obtained from Monte Carlo permutation tests with 10,000 repetitions.

^d^SNAP-IV: Swanson, Nolan, and Pelham questionnaire.

^e^ODD: oppositional defiant disorder.

^f^BRIEF: Behavior Rating Inventory of Executive Function.

^g^BRI: Behavior Regulation Index.

^h^MI: Metacognition Index.

^i^GEC: Global Executive Composite.

## Discussion

### Principal Findings

This study investigated one of the first digital interventions combining both cognitive and physical (exercise) training to address the core symptoms of ADHD and EF deficits. Compared to those in the control group, children randomized to the BrainFit intervention showed statistically significant improvement in reducing symptoms of ADHD and enhancing EF, as rated by their caregivers.

To our knowledge, this is the first study combining cognitive training and exercise therapy, two effective interventions for elementary and middle school–aged children with ADHD. Findings from this study provide evidence that a digital cognitive-physical intervention could be a promising nonpharmacologic treatment for ADHD.

The therapeutic effects of BrainFit are comparable to those reported in previous studies. A recent systematic review and meta-analysis of 14 trials reported that digital interventions significantly improved attention of children with ADHD (SMD 0.28, 95% CI 0.14-0.41). Our study demonstrated an improved inattention score with a comparable effect (SMD 0.986, 95% CI 0.581-01.447). This evidence suggested a comparable or even greater improvement of inattention than previous digital interventions, which could be related to the application of the exercise intervention. However, our intervention showed disadvantages in certain subdomains of ADHD symptoms. Davis et al [41] reported that the digital intervention “EVO” could improve attention, working memory, and inhibition in children with ADHD. However, in this study, the scores on the Inhibit, Shift, and Emotional Control subscales did not differ significantly between the intervention and control groups, which could be related to the design of the intervention or subjectivity of caregiver ratings.

With the development of digital technologies [42], increasing studies have demonstrated their promising role in the management of various diseases, especially mental and behavioral disorders [43]. Compared with traditional pharmacotherapy and behavioral interventions, digital interventions have the advantages of a minimal requirement of human resources and less adverse effects, which could reach more patients worldwide, especially for patients in less-developed countries and regions. With greater accessibility and compliance, digital therapies could provide proper and timely intervention for children with ADHD, reducing the number of children who do not receive any intervention to manage their symptoms. The US FDA approved a game-based digital therapy called “EndeavorRx” for the treatment of ADHD on June 15, 2020, which demonstrated therapeutic effects on attention among children aged 8-12 years with ADHD [44]. Emerging digital therapies such as BrainFit have the potential to be translated to clinical settings, thereby offering clinicians and families more choices for ADHD intervention.

Cognitive training has been shown to reduce deficits in EF in children with ADHD by strengthening neural networks, while physical exercise increases the levels of catecholamines such as dopamine and norepinephrine [17,27,45,46]. Exercise that is cognitively engaging can increase cognitive abilities in children with ADHD by engaging the regions of the brain involved in EF [16,27]. An “exergame” developed by Benzing and Schmidt [25] was tested in 51 children (aged 8-12 years) in a randomized controlled study that spanned 8 weeks. The game employed strength, coordination, endurance, attention, inhibition, and task-shifting. The treatment group showed improved attention, inhibition, task-shifting, general mental health, and motor abilities compared with those of the control group [25].

In another study, 35 adolescents were assigned to a treatment arm of ADHD medication and 6 weeks of exercise consisting of a sports program or to a control group of ADHD medication and education, and the results showed that the addition of exercise had a strong influence on task-shifting and improving the speed of attention processing [47]. The exercise program consisted of approximately 90-minute sessions three times per week in which there was a 10-minute warm-up period; a 60-minute aerobic exercise that engaged EF such as zigzag running, jumping rope individually and in groups, and basketball; followed by a 10-minute cool-down period [47]. It is thus recommended that game content should be included in any intervention program prescribing exercise, and the exercises should be planned and combined [27,46]. Intensity should be emphasized, while perceptual motor exercises should be combined with cognitive training such as motor planning [16,27]. This strategy has been shown to improve social, perceptual, and cognitive functions in children with ADHD [27,46].

### Limitations

Our study showed promising results for a novel digital cognitive-physical intervention targeting symptoms of ADHD and EF deficits. However, several limitations should be considered in interpreting the findings. First, this was a single-center study with a limited sample size; thus, the study was not powered for subscale analyses across measures. Second, the generalizability of our study findings is limited by our lack of control for ADHD-medication use, ADHD subtype, and age distribution in patients with ADHD. Third, this study measured the outcomes immediately after the conclusion of the 4-week intervention; thus, we are unable to assess the durability of the treatment effects. Based on the encouraging findings of this study, a multicenter efficacy trial controlling for medication use and ADHD subtype with a broader age distribution and a longer follow-up period is planned. Fourth, a dose-finding study with different frequencies of sessions may be necessary to optimize the intervention dose and assess treatment heterogeneity. Finally, the lack of blinding and a sham control may have led to expectancy effects from the parents in completing the assessments.

### Conclusion

The findings of this randomized controlled trial conducted in China suggest that BrainFit, a novel digital intervention combining cognitive training and exercise, could improve the symptoms and EF in children with ADHD. The intervention has the potential to increase access to ADHD treatment and improve ADHD symptoms in school-aged children.

## Appendix

Multimedia Appendix 1 CONSORT-eHEALTH checklist (V 1.6.1).

## Abbreviations

ADHD: attention-deficit/hyperactivity disorder
AR: augmented reality
BRI: Behavioral Regulation Index
BRIEF: Behavior Rating Inventory of Executive Function
CONSORT: Consolidated Standards for Reporting Trials
DSM-5: Diagnostic and Statistical Manual of Mental Disorders, Fifth Edition
EF: executive function
FDA: Food and Drug Administration
GEC: Global Executive Composite
MI: Metacognition Index
ODD: oppositional defiant disorder
SMD: standardized mean difference
SNAP-IV: Swanson, Nolan, and Pelham questionnaire

## Data Availability

The data sets generated and used in the current study are available from the corresponding author on reasonable request.
